# Direct Visualization of Acute Left Atrial Pressure Elevation During Atrioventricular Nodal Re-Entrant Tachycardia

**DOI:** 10.1016/j.jaccas.2026.109879

**Published:** 2026-08-18

**Authors:** Tae Kyung Yoo, Caroline Kaufman, David R. Pimentel, Tiffany Y. Hu, Sebastian Rodriguez, Paul Franco, Eric H. Awtry, Ashvin N. Pande, Deepa M. Gopal, Nir Ayalon

**Affiliations:** a Cardiovascular Medicine Section, Department of Medicine, Boston Medical Center, Boston University Chobanian & Avedisian School of Medicine, Boston, Massachusetts, USA; b Department of Medicine, Boston Medical Center, Boston, Massachusetts, USA

**Keywords:** acute heart failure, hemodynamics, remote monitoring, supraventricular arrhythmias

## Abstract

**Background:**

Supraventricular tachycardia (SVT) can acutely elevate left atrial pressure (LAP), yet real-time hemodynamic data during spontaneous episodes are rarely captured. We present a case of real-time capture of LAP during SVT episodes using the implantable V-LAP system (Vectorious Medical Technologies), a new LAP monitoring sensor implanted in the interatrial septum of patients with heart failure.

**Case Summary:**

A 55-year-old female patients with heart failure and an implanted implantable left atrial pressure (V-LAP) sensor presented with symptomatic SVT, which terminated with intravenous adenosine. The V-LAP sensor revealed that LAP rose acutely from 4.4 mm Hg to 16.4 mm Hg during SVT, returning to 3.2 mm Hg after its termination. An electrophysiology study confirmed typical atrioventricular nodal reentrant tachycardia, and successful ablation was performed.

**Discussion:**

This case provides the first direct visualization of elevated LAP during an atrioventricular nodal reentrant tachycardia episode and suggests that typical atrioventricular nodal reentrant tachycardia ablation was feasible, without device interference, in this patient with a V-LAP sensor. This case highlights the value of integrated electrical and hemodynamic monitoring in managing patients with cardiomyopathy and arrhythmia.

**Take-Home Messages:**

The V-LAP sensor supports the hemodynamic contribution to symptom generation by correlating real-time LAP elevation with arrhythmia. Additionally, standard atrioventricular nodal reentrant tachycardia ablation was feasible in this patient with an interatrial septal sensor, without device interference.

## Visual Summary

**Figure:**
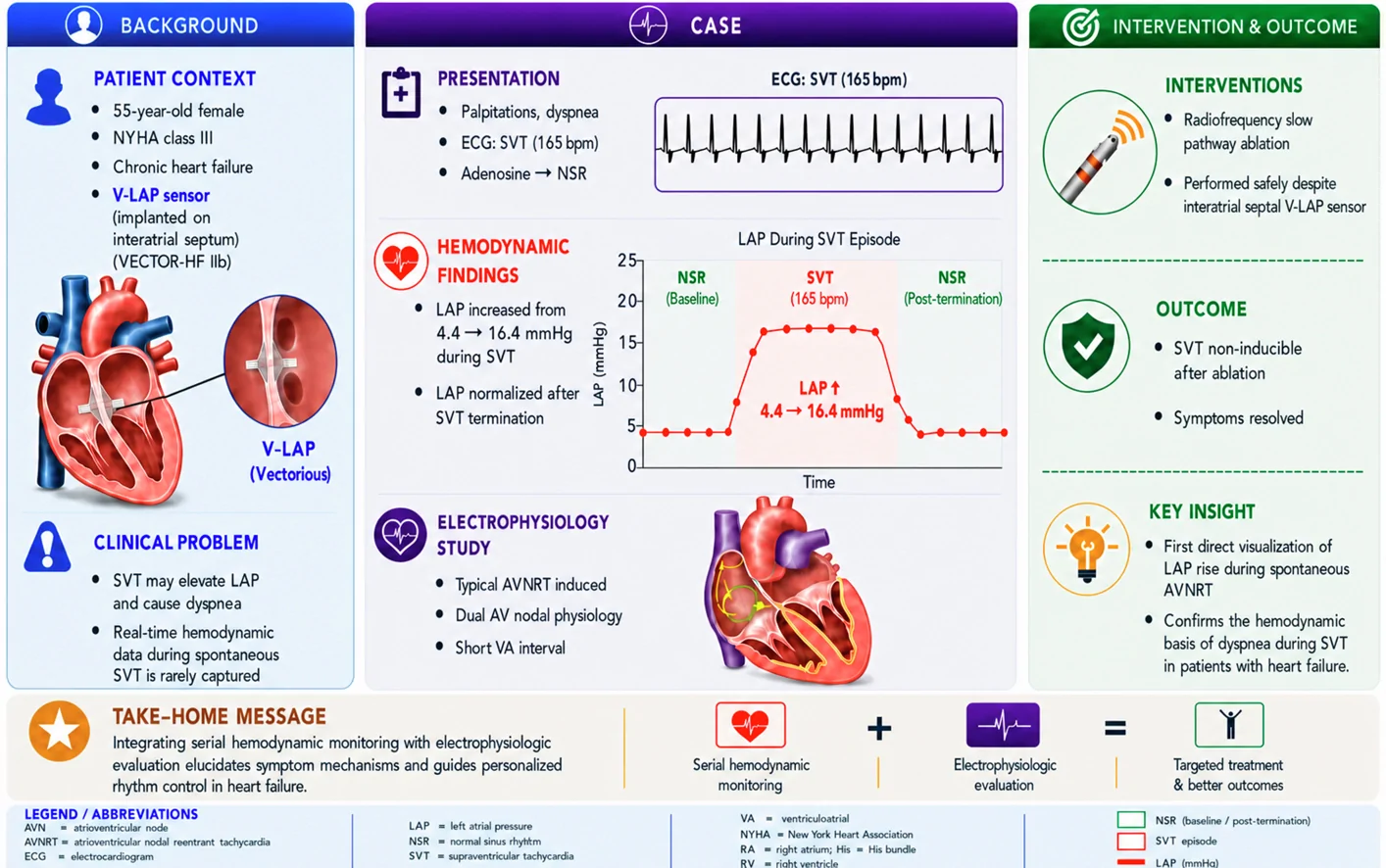

## History of Presentation

A 55-year-old female patients presented to the emergency department with acute-onset palpitations and dyspnea. She was in her usual state of health until the morning of presentation, when she developed sudden, severe palpitations, along with shortness of breath. Initially, she was tachycardic to 160 beats/min, blood pressure 91/56 mm Hg, respiratory rate 30/hr, SpO_2_ 96% on room air, and body temperature 98.1°F. The patient remained alert and oriented. Physical exam showed tachycardic but regular heart rhythm, elevated jugular venous pressure. An initial electrocardiogram demonstrated a regular, narrow QRS complex tachycardia (Figure 1).

**Figure 1:**
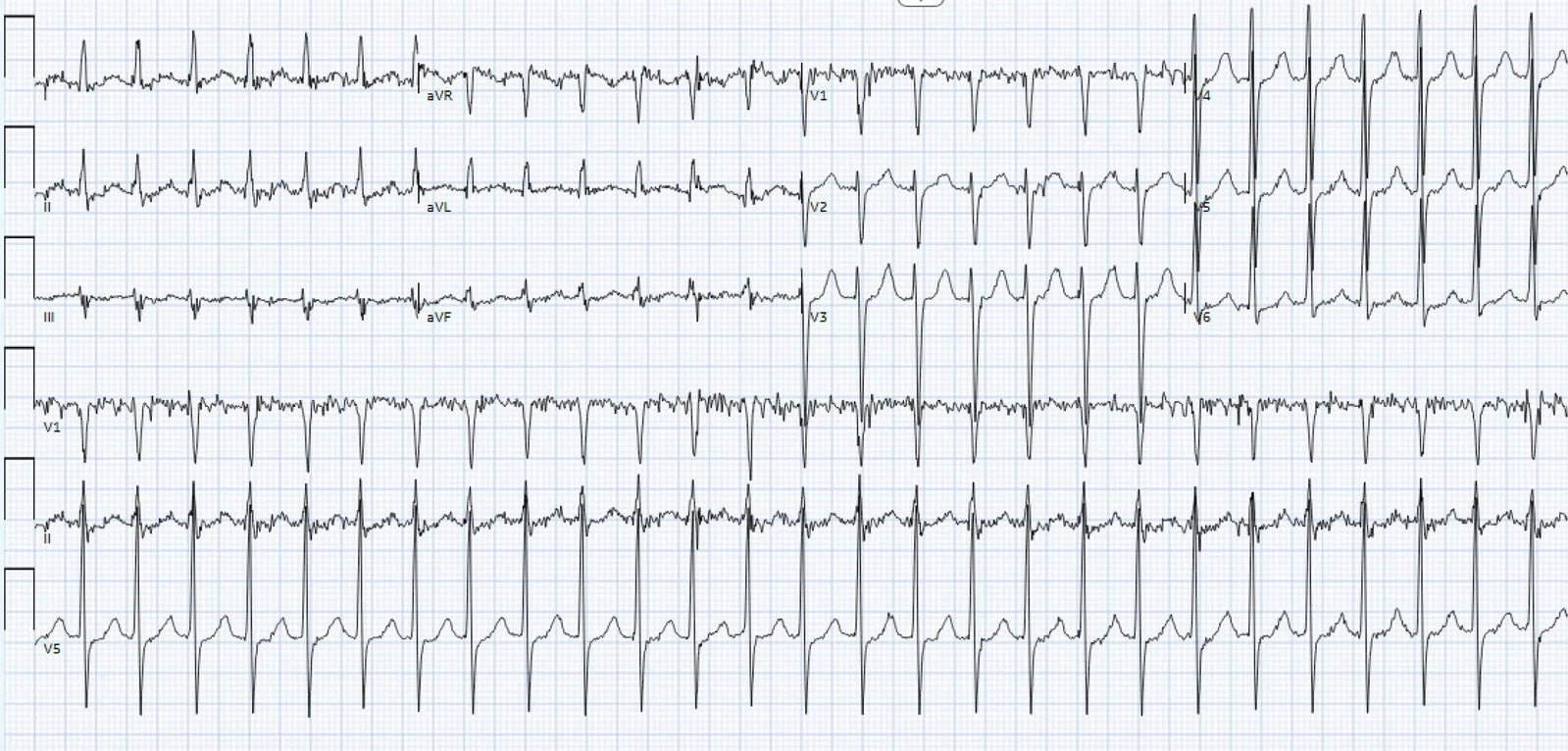
Initial Electrocardiogram on Presentation Heart rate 165 beats/min, corrected QTc 483 ms; aVF = augmented vector foot; aVL = augmented vector left; aVR = augmented vector right.

## Past Medical History

The patient was enrolled in the VECTOR-HF (V-LAP Left Atrium Monitoring systEm for Patients With Chronic sysTOlic & Diastolic Congestive heaRt Failure) IIb trial. The study design was described in detail in a previous paper. Briefly, the V-LAP system is a battery-free, wireless pressure sensor permanently implanted in the interatrial septum via a transseptal approach. Daily left atrial pressure (LAP) recordings are acquired using an external wearable belt and transmitted to a cloud-based interface. The sensor is passive and stores no data on its own; it is energized and interrogated only when the patient activates an external reader, so each recording is a discrete, patient-initiated transmission of a brief high-resolution LAP waveform. These data drive a physician-directed patient self-management application, allowing patients to adjust diuretic therapy based on real-time hemodynamic ranges. Inclusion criteria included adults with chronic HF, NYHA functional class III status, and a history of worsening HF despite optimal therapy; the current patient met these requirements.^1^^,^^2^ In addition, she had a primary prevention single-chamber implantable cardioverter-defibrillator (ICD, Medtronic), hypertension, hyperlipidemia, and was on chronic anticoagulation because of prior pulmonary embolism. Her home cardiac medications included apixaban 5 mg twice daily, empagliflozin 10 mg daily, losartan 25 mg daily, metoprolol succinate 25 mg daily, and torsemide 10 mg daily. She had been independently adjusting her home torsemide dosing based on LAP sensor readings.

## Differential Diagnosis

Given the patient’s structural heart disease and history of LAP sensor implantation, the initial differential diagnosis primarily included focal or macro-reentrant atrial tachycardia and atypical atrial flutter. However, based on the electrocardiogram findings, atrioventricular nodal reentrant tachycardia (AVNRT) emerged as the most likely diagnosis, though atrioventricular reentrant tachycardia was also considered.

## Investigations

Laboratory evaluation was largely unremarkable, including a white blood cell count of 6.7 K/μL and hemoglobin of 12.7 g/dL. Electrolyte level were normal (sodium 143 mmol/L, magnesium 1.9 mg/dL, calcium 9.3 mg/dL), with the exception of mild hypokalemia (potassium 3.4 mmol/L). Renal function showed a creatinine of 1.2 mg/dL. High-sensitivity troponin was negative (<3 ng/L), and lactate was 2.1 mmol/L.

Transthoracic echocardiography performed 2 weeks before admission revealed a normal left ventricular size with reduced systolic function (left ventricular ejection fraction 30%-35%) and global hypokinesis. Right ventricular systolic function was normal, and mild mitral regurgitation was noted. Point-of-care ultrasound at the time of admission revealed no pericardial effusion, and an estimated left ventricular ejection fraction of 30%. A chest radiograph demonstrated clear lung fields without pulmonary opacities, with a normal cardiac silhouette. ICD interrogation demonstrated 7 supraventricular tachycardia (SVT) episodes on the day of presentation, with an average ventricular rate of 182 beats/min. Investigation of her implanted LAP sensor demonstrated an elevated mean LAP of 16.4 mm Hg during the symptomatic, patient-initiated transmission, compared with 4.4 mm Hg on her routine measurement earlier that morning, with normalization to 3.2 mm Hg on a subsequent transmission after termination of the arrhythmia (Figure 2).

**Figure 2:**
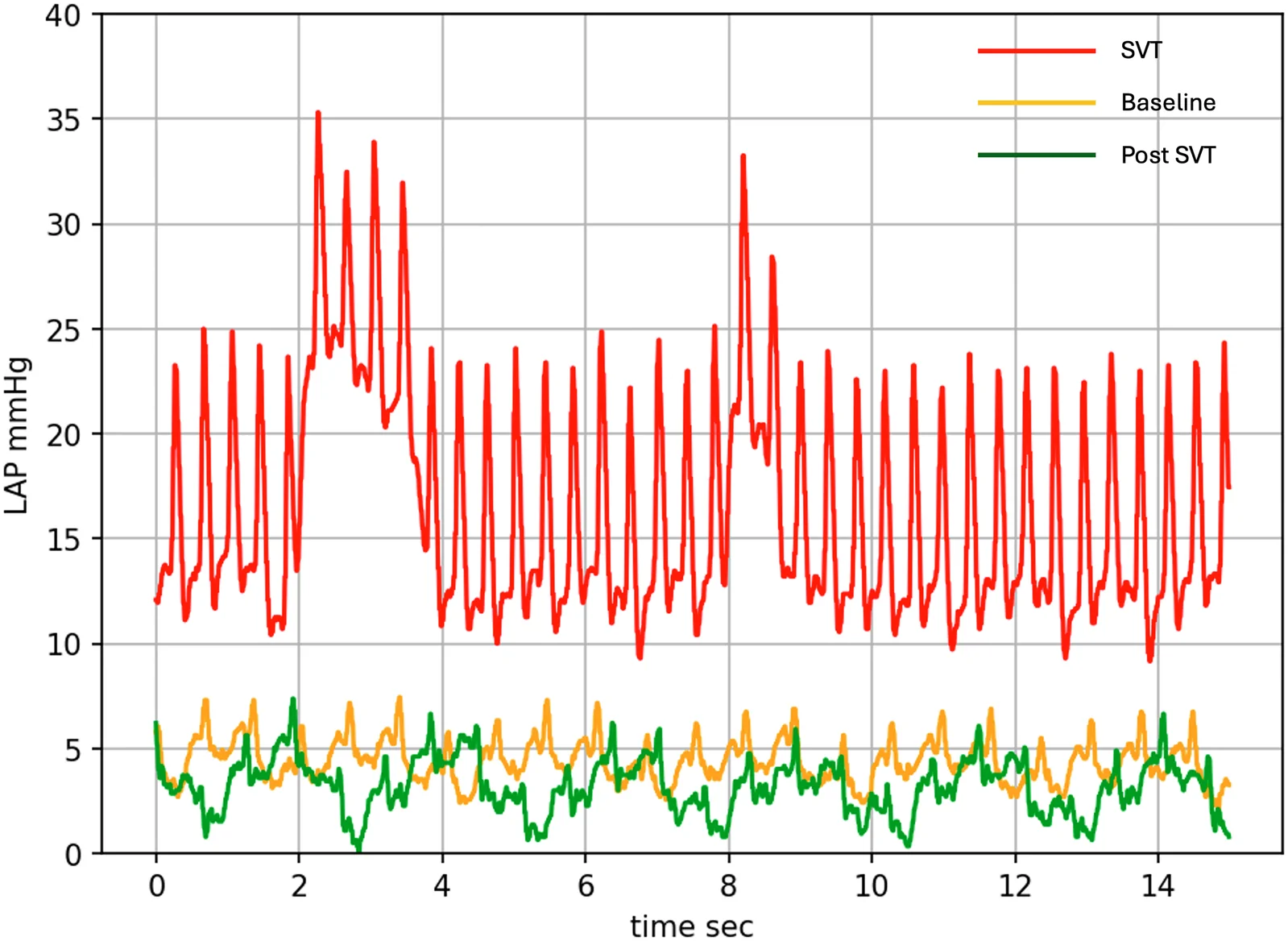
Left Atrial Tracing From Implantable Left Atrial Pressure Sensor Sensor During Spontaneous Supraventricular Tachycardia Episode The left atrial pressure (LAP) waveform from the symptomatic transmission; no simultaneous home electrocardiogram was available, so alignment with the rhythm is based on the temporal coincidence of her symptoms, the transmitted pressure elevation, and the subsequently documented supraventricular tachycardia (SVT).

## Management

Initial management to abort her SVT with vagal maneuvers was unsuccessful. She then received adenosine 6 mg intravenously, with successful termination of the arrhythmia back to normal sinus rhythm. Given the hemodynamic effects and significant symptomatic burden, an electrophysiologic (EP) study and catheter ablation were planned. The presence of the interatrial septal LAP sensor was carefully considered, particularly if transseptal access was required.

She underwent a comprehensive EP study with isoproterenol infusion. The procedure used standard femoral venous access with quadripolar catheters at the high right atrium, His position, and right ventricle, a decapolar coronary sinus catheter, and 3-dimensional electroanatomic mapping. Dual atrioventricular (AV) nodal physiology was present, and typical AVNRT was readily induced with programmed atrial stimulation. The septal ventriculoatrial (VA) time was short (26 ms), consistent with near-simultaneous VA activation (Figure 3). Atrial tachycardia was excluded by a V-A-V response following ventricular entrainment, and atrioventricular reentrant tachycardia was excluded by a long postpacing interval minus tachycardia cycle length (of 250 ms) and by para-Hisian pacing demonstrating a nodal response, excluding a septal accessory pathway. Radiofrequency ablation of the slow pathway was successfully performed using a 4-mm ablation catheter at the inferior aspect of the coronary sinus ostium. Because typical AVNRT ablation is performed entirely from the right atrium, no transseptal puncture was required and the septal sensor was not instrumented. Following ablation, SVT was no longer inducible despite programmed stimulation, with and without isoproterenol. The patient tolerated the procedure well without acute complications.

**Figure 3:**
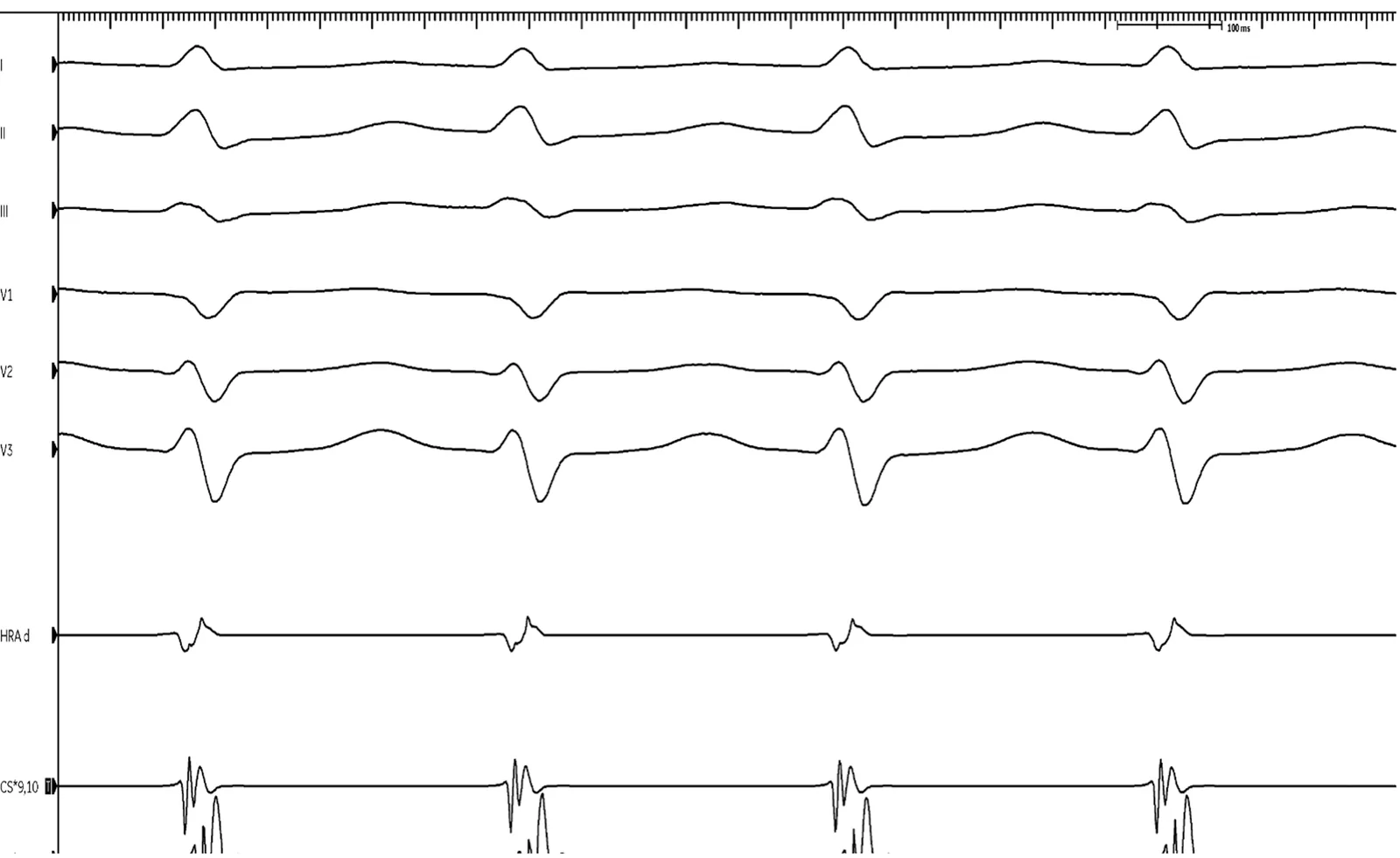
Electrogram During Electrophysiology Study Electrogram during the electrophysiology study demonstrating septal ventriculoatrial activation time of 26 ms, consistent with near-simultaneous ventriculoatrial activation suggesting atrioventricular nodal reentrant tachycardia.

## Outcomes/Follow-Up

The patient remained in sinus rhythm postprocedure, and had been asymptomatic, with no recurrence of palpitations, dyspnea, or hypotension (Figure 4). Ongoing follow-up was arranged, with continued LAP-guided diuretic management. At the 3-month postdischarge follow-up clinic visit, interrogation of her ICD revealed no new SVT episodes.

**Figure 4:**
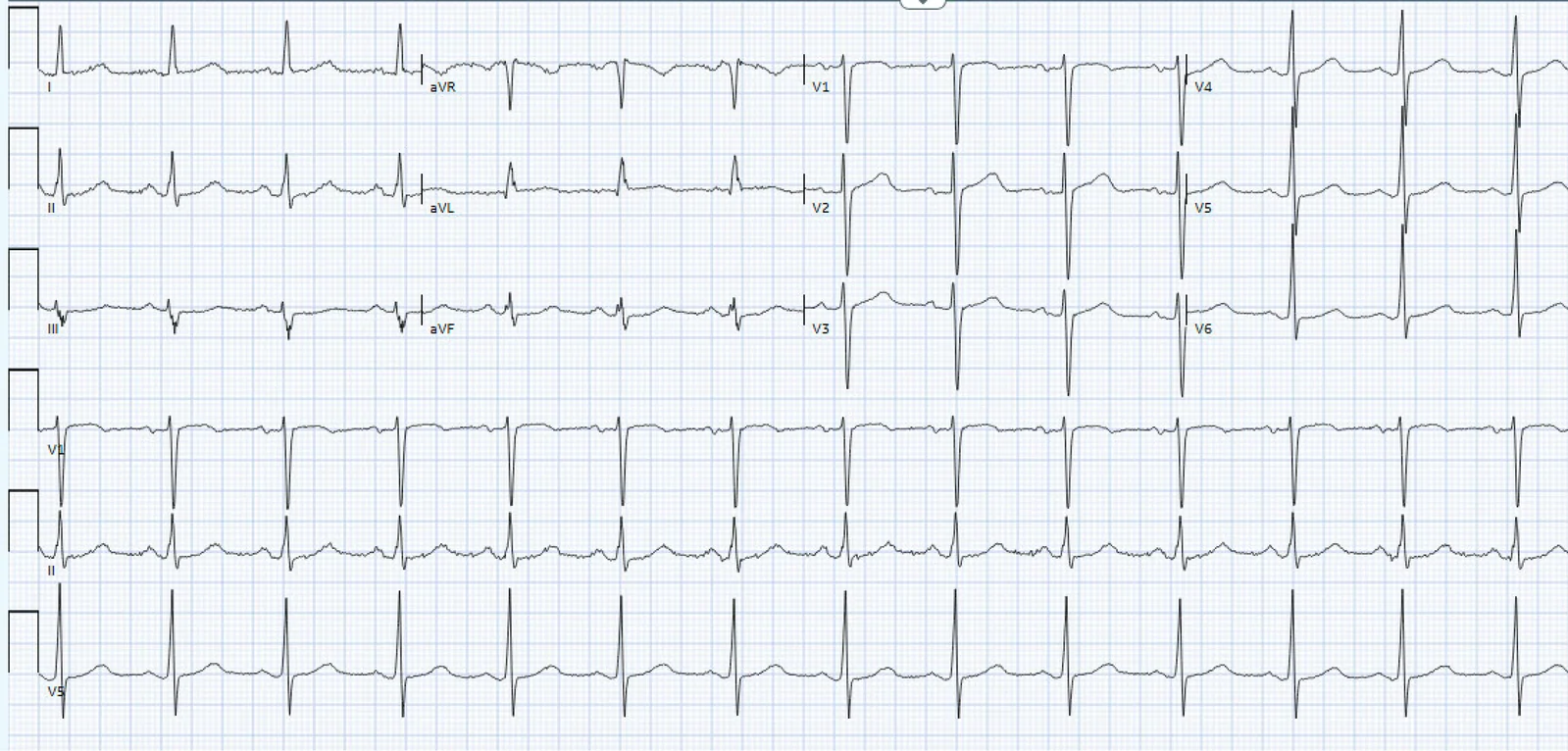
Electrocardiogram Before Discharge Normal sinus rhythm, heart rate 82 beats/min, QTc 464 ms; other abbreviations as in Figure 1.

## Discussion

SVT can significantly impair cardiac hemodynamic function by shortening diastolic filling time, disrupting atrioventricular synchrony, and acutely elevating LAP.^3^ These effects are magnified in patients with underlying systolic dysfunction and elevated baseline filling pressures. In this patient with NYHA functional class III HF due to nonischemic cardiomyopathy, the abrupt onset of AVNRT produced marked dyspnea and transient hypotension, consistent with a sudden increase in left-sided filling pressures and impaired forward cardiac output. These symptoms were most likely multifactorial—the combined result of the rapid ventricular rate, loss of AV synchrony, shortened diastolic filling, and reduced forward output—with the acute rise in LAP representing 1 measurable manifestation of these interrelated effects rather than the sole cause.

Although the hemodynamic consequences of tachyarrhythmia in HF are well described in the literature, direct real-time documentation of LAP changes during spontaneous SVT has been limited. This case provides the first direct visualization of acute LAP elevation during spontaneous AVNRT using an implanted interatrial septal pressure sensor. The close temporal relationship between arrhythmia onset and a rapid rise in LAP, followed by a normalization after termination of SVT, offers mechanistic support for symptom generation and illustrates the vulnerability of patients with advanced HF to arrhythmia. Because each recording is a brief patient-initiated transmission, we cannot determine how long the arrhythmia had been present before the measurement; whereas a longer period of AV dyssynchrony could in principle permit greater accumulation of LAP, our single-timepoint data cannot establish a duration–magnitude relationship.

Catheter ablation is a class I recommended therapy for symptomatic AVNRT and is particularly favored in patients with either recurrent episodes or intolerance to medical management.^4^^,^^5^ In patients with concurrent HF, restoration of atrioventricular synchrony and prevention of recurrent tachycardia may confer disproportionate symptomatic benefit. In this case, the patient's high symptomatic burden and hemodynamic instability during SVT supported early definitive treatment with catheter ablation.

A unique aspect of this case is the coexistence of an interatrial septal LAP sensor, which raised procedural considerations should transseptal access be required. Although typical AVNRT ablation is performed from the right atrium and does not routinely require transseptal puncture, careful preprocedural planning was warranted given the presence of her septal implant. Slow pathway ablation was feasible in this patient and was performed without interference with the LAP sensor. A single case cannot establish broader safety, but it suggests feasibility when device position is accounted for in procedural planning.

Beyond its EP implications, this case highlights the emerging role of combined electrical and hemodynamic monitoring in patients with cardiomyopathy. Serial LAP monitoring provides unique physiologic insight into symptom causation and may serve as a valuable adjunct for risk stratification and therapeutic decision-making in patients with both HF and arrhythmia. As implantable hemodynamic sensors become more widely adopted, their integration into arrhythmia evaluation may further refine patient selection for invasive therapies and enhance understanding of cardio-hemodynamic interactions.

## Conclusions

This case provides the first direct, real-time hemodynamic demonstration of acute LAP elevation during spontaneous AVNRT in a patient with HF, confirming the arrhythmia's role in symptom generation for patients with HF. Furthermore, this case demonstrates that standard slow pathway ablation in AVNRT was feasible in this patient with an indwelling interatrial pressure sensor, without device interference.

**Visual Summary:**
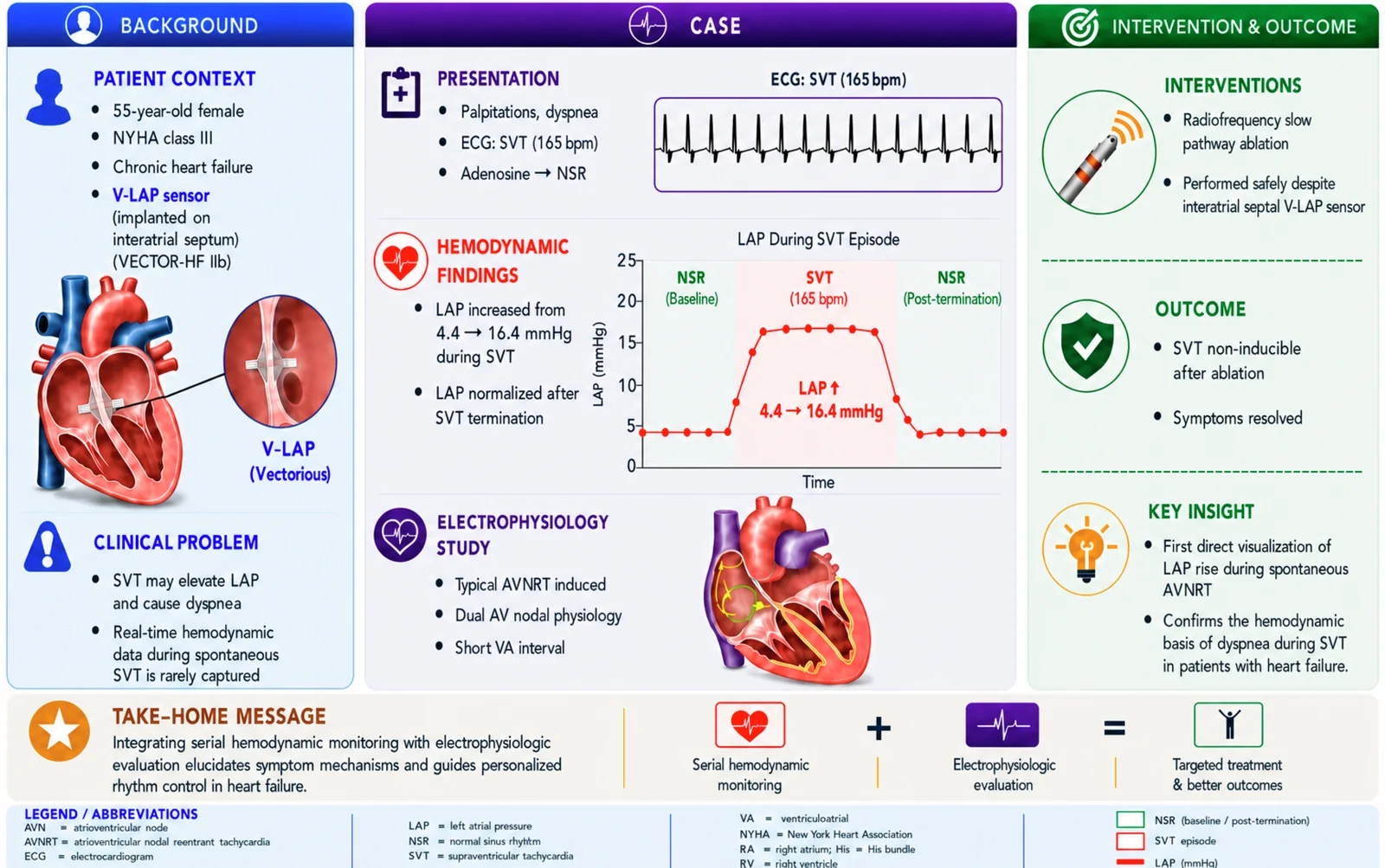
Spontaneous AVNRT in a Patient With Chronic HF and an Implanted Investigational LAP Sensor (V-LAP, Vectorious Medical Technologies Ltd) Caused an Acute, Symptomatic Rise in LAP (4.4-16.4 mm Hg), Which Normalized After Rhythm Restoration With Adenosine, Resulting in Symptom Resolution Electrophysiologic study confirmed typical AVNRT, and successful slow-pathway ablation eliminated arrhythmia recurrence. AV = atrioventricular ; AVN = atrioventricular nodal; ECG = electrocardiogram; NSR = normal sinus rhythm; RA = right artium; RV = right ventricle; SVT = supraventricular tachycardia; VA = ventriculoatrial; VECTOR-HF IIb = V-LAP Left Atrium Monitoring systEm for Patients With Chronic sysTOlic & Diastolic Congestive heaRt Failure; V-LAP = implantable left atrial pressure.

## Funding Support and Author Disclosures

This case report was prepared independently and was not supported by dedicated funding. The patient described in this report was enrolled in the multicenter, international VECTOR-HF IIb study, sponsored by Vectorious Medical Technologies, Tel Aviv, Israel. The corresponding author, Dr Ayalon, serves as a site Principal Investigator for the VECTOR-HF IIb study and receives research support from Vectorious Medical Technologies for the conduct of the trial at his institution. The sponsor had no role in the analysis or interpretation of the data, the writing of this manuscript, or the decision to submit it for publication. Dr Ayalon receives consulting fees from Abiomed, Alleviant, Breeoot, Restore, and Vectorious; and holds shares in Breeoot. All other authors have reported that they have no relationships relevant to the contents of this paper to disclose.

**Take-Home Messages**

• This case highlights the clinical value of integrating serial hemodynamic data with electrophysiologic evaluation in patients with cardiomyopathy and arrhythmia.
• Such multimodal monitoring may enhance mechanistic understanding, support guideline-directed rhythm control strategies, and inform future approaches to personalized arrhythmia management in HF populations.
